# Documented Firearm Access Before Suicide Among Psychiatric Emergency Service Patients

**DOI:** 10.1001/jamanetworkopen.2025.20017

**Published:** 2025-07-11

**Authors:** Anne E. Massey, Paul R. Borghesani, Jennifer Stuber, Frederick P. Rivara, Ali Rowhani-Rahbar

**Affiliations:** 1 Department of Epidemiology, School of Public Health, University of Washington, Seattle; 2 Firearm Injury and Policy Research Program, School of Medicine, University of Washington, Seattle; 3 Department of Psychiatry & Behavioral Sciences, School of Medicine, University of Washington, Seattle; 4 Harborview Medical Center, University of Washington, Seattle; 5 School of Social Work, University of Washington, Seattle; 6 Department of Pediatrics, School of Medicine, University of Washington, Seattle

## Abstract

This case series evaluates firearm access among patients assessed in the psychiatric emergency service who died by suicide.

## Introduction

Firearms are used in over 50% of all suicide deaths in the US.^1^ Reducing firearm access among people who may be at risk of suicide is strongly recommended (eg, safe storage).^2^ Health care encounters offer a unique opportunity to assess firearm access and counsel as needed.^3^ Accordingly, a large US health care system added a firearm access question to their electronic health record (EHR).^4^ They subsequently found that over 50% of patients who answered the question during an ambulatory care encounter and later died by firearm suicide reported no firearm access at their visit. We conducted a similar study in an alternate clinical setting and hypothesized that a comparable proportion of patients who died by firearm suicide would report no firearm access prior to death.

## Methods

In this case series, we linked patients who were assessed in the psychiatric emergency service (PES) in an urban county hospital in Washington state from January 1, 2012, through December 31, 2017 (n = 15 652), with Washington state death records from January 1, 2012, through December 31, 2021 (n = 566 819). We determined case status using *International Statistical Classification of Diseases and Related Health Problems, Tenth Revision* codes (eTable 1 in Supplement 1) and firearm access using an algorithm composed of 3 discrete variables (eTable 2 in Supplement 1) from a standardized, EHR-embedded suicide risk assessment used during every visit. We reported documented firearm access at patients’ last visit before death and stratified findings by deaths that occurred within 1 year of patients’ last visit and over 1 year later. The Washington State Institutional Review Board approved this study and granted a waiver of informed consent after determining the study was minimal risk. Analyses were conducted from July to November 2024 using The Link King software and R, version 4.4.1. Results reporting adhered to the STROBE reporting guideline.

## Results

We identified 181 patients who died by suicide (mean [SD] age at last PES visit, 39 [14] years; 63 females [34.8%] and 118 males [65.2%]). Twenty-nine suicide deaths (16.0%) were by firearm. Compared with people who died by nonfirearm suicide (n = 152), people who died by firearm suicide were more likely to be male, White, and non-Hispanic (Table). Documented firearm access was nearly 20 times higher among people who died by firearm suicide than among people who died by nonfirearm suicide (13.8% [4 of 29] vs 0.7% [1 of 152]). The higher proportion of documented access among people who died by firearm suicide persisted when stratified by time between patients’ last visit and death (Figure). Fourteen people who died by firearm suicide (48.3%) had documentation indicating they reported not having access to a firearm at their last visit. Unknown firearm access was similar between those who died by firearm (37.9% [11 of 29]) and nonfirearm means (32.9% [50 of 152]).

**Table.** Demographic Characteristics Among PES Patients Who Died by Suicide

Characteristic	Patients, No. (%)		
	Firearm suicide (n = 29)	Nonfirearm suicide (n = 152)	All (N = 181)
Age at last PES visit before death, mean (SD) [range], y	39 (15) [18-71]	39 (14) [14-76]	39 (14) [14-76]
Gender			
Female	9 (31.0)	54 (35.5)	63 (34.8)
Male	20 (69.0)	98 (64.5)	118 (65.2)
Race^a^^,^^b^			
American Indian or Alaska Native	0	3 (2.0)	3 (1.7)
Asian^c^	3 (10.3)	13 (8.6)	16 (8.8)
Black or African American^d^	1 (3.4)	19 (12.5)	20 (11.0)
Mexican, Mexican-American, or Chicano	0	1 (0.7)	1 (0.6)
Middle Eastern	1 (3.4)	0	1 (0.6)
Native Hawaiian or Other Pacific Islander	0	1 (0.7)	1 (0.6)
White or Caucasian^e^	24 (82.8)	114 (75.0)	138 (76.2)
Unknown	0	1 (0.7)	1 (0.6)
Ethnicity^a^			
Hispanic	0	4 (2.6)	4 (2.2)
Non-Hispanic	24 (82.8)	122 (80.3)	146 (80.7)
Unknown^f^	5 (17.2)	26 (17.1)	31 (17.1)
State of residence			
Washington	28 (96.6)	147 (96.7)	175 (96.7)
Other	1 (3.4)	5 (3.3)	6 (3.3)
Time from last PES visit to death, No. of patients			
≤1 y	11	65	76
>1 y	18	87	105

Abbreviations: EHR, electronic health record; PES, psychiatric emergency service.

^a^ Because suicide rates vary by race and ethnicity,^1^ we assessed both in this study. Race and ethnicity were self-reported, obtained from the EHR, and separate fields in the EHR.

^b^ These are the categories as they appeared in the EHR. Due to the changing nature of the EHR over time, we do not have a comprehensive list of all available race categories across the entirety of our study, only those that were documented in our sample.

^c^ There was 1 patient who died by nonfirearm suicide whose race was documented as Vietnamese. For ease of presentation, we collapsed this category with the Asian category.

^d^ There was 1 patient who died by firearm suicide and 7 patients who died by nonfirearm suicide whose race was documented as Black. For ease of presentation, we collapsed this category with the Black or African American category.

^e^ There were 8 patients who died by firearm suicide and 43 patients who died by nonfirearm suicide whose race was documented as Caucasian. For ease of presentation, we collapsed this category with the White category.

^f^ Unknown includes a formal EHR category for unknown or unreported ethnicity, as well as any patient who died by suicide for whom a response to the ethnicity field was missing (4 patients who died by firearm suicide and 15 patients who died by nonfirearm suicide).

**Figure.**
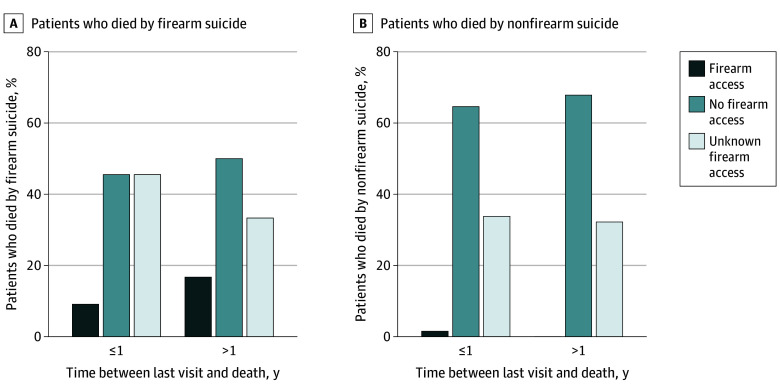
Documented Firearm Access at the Last Psychiatric Emergency Service Visit Before Death Among Patients Who Died by Suicide

## Discussion

Similar to the prior study,^4^ we found that approximately half of all patients who died by firearm suicide had documentation indicating they did not have firearm access at their last visit before death. Although they may have acquired firearms after their visit, it is also possible some had access they did not disclose due to anticipated stigma or other concerns (eg, privacy, firearm removal).^5^ We also found that about one-third of all patients who died by suicide had unknown access. Both findings suggest a need for more effective strategies regarding how clinicians discuss firearms and firearm safety with patients and their families. Approaches may include further leveraging the EHR (eg, mandating relevant fields) and evidence-based training to increase clinicians’ comfort and confidence discussing firearms.^6^ Further research is needed to assess what strategies will be most effective. Given the uniqueness of the PES setting, a limitation of our study is that our findings may have limited generalizability. Additional efforts are necessary to better understand firearm access in this population and identify strategies that meaningfully support clinicians discussing firearm safety with PES patients specifically.

## Notes

Supplement 1. **eTable 1.** *ICD-10* Codes Used to Identify Cases **eTable 2.** Firearm Access Classification Algorithm

Supplement 2.
Data Sharing Statement
