# Supplementary material for: Documented Firearm Access Before Suicide Among Psychiatric Emergency Service Patients
Source: JAMA Netw Open. 2025 Jul 11;8(7):e2520017. doi: 10.1001/jamanetworkopen.2025.20017 (PMC12254887; doi:10.1001/jamanetworkopen.2025.20017)
Supplement: Supplement 1. — eTable 1. ICD-10 Codes Used to Identify Cases eTable 2. Firearm Access Classification Algorithm [file jamanetwopen-e2520017-s001.pdf]

Supplementary Online Content

Massey AE, Borghesani PR, Stuber J, Rivara FP, Rowhani-Rahbar A. Documented firearm access before suicide among psychiatric emergency service patients. *JAMA Netw Open.* 2025;8(7):e2520017. doi:10.1001/jamanetworkopen.2025.20017

eTable 1. *ICD-10* Codes Used to Identify Cases
eTable 2. Firearm Access Classification Algorithm

This supplementary material has been provided by the authors to give readers additional information about their work.

eTable 1. *ICD-10* Codes Used to Identify Cases

Outcome	ICD-10 Codes
**Firearm Suicide**	X72-X74
**Non-Firearm Suicide**	X60-X71, X75-X84, Y87.0, U03

© 2025 Massey AE et al. *JAMA Network Open.*

eTable 2. Firearm Access Classification Algorithm

Exposure	Suicide Risk Assessment Variables
**Firearm Access**	Firearms = “Yes” **OR**<br><br>Firearms and pills = “Yes”
**No Firearm Access**	Access to lethal means = “No” **OR**<br><br>Firearms = “No” **AND** Firearms and pills = “No”
**Unknown Firearm Access**	Firearms = blank **AND**<br><br>Firearms and pills = blank

© 2025 Massey AE et al. *JAMA Network Open.*
