# Supplementary material for: Documented Firearm Access Before Suicide Among Psychiatric Emergency Service Patients
Source: JAMA Netw Open. 2025 Jul 11;8(7):e2520017. doi: 10.1001/jamanetworkopen.2025.20017 (PMC12254887; doi:10.1001/jamanetworkopen.2025.20017)
Supplement: Supplement 2. — Data Sharing Statement [file jamanetwopen-e2520017-s002.pdf]

Data Sharing Statement

Massey. Documented Firearm Access Before Suicide Among Psychiatric Emergency Service Patients. *JAMA Netw Open*. Published July 11, 2025.
doi:10.1001/jamanetworkopen.2025.20017

Data
Data available: No
